# Efficacy and safety of DEB-TACE combined with transarterial TQB2450 infusion and oral Anlotinib as first-line treatment in advanced hepatocellular carcinoma: a single-arm phase II study

**DOI:** 10.3389/fimmu.2025.1667785

**Published:** 2025-09-01

**Authors:** Hang Yuan, Yan Li, Ke Zhao, Ya-Nan Zhao, Quan-Jun Yao, Xiang Geng, Li-Feng Wang, Ho-Young Song, Hong-Tao Hu

**Affiliations:** ^1^ Department of Interventional Radiology, The Affiliated Cancer Hospital of Zhengzhou University & Henan Cancer Hospital, Zhengzhou, China; ^2^ Department of Radiology, The Affiliated Cancer Hospital of Zhengzhou University & Henan Cancer Hospital, Zhengzhou, China

**Keywords:** hepatocellular carcinoma, DEB-TACE, TQB2450, Anlotinib, PD-L1 inhibitor

## Abstract

**Purpose:**

Advanced hepatocellular carcinoma (HCC) remains difficult to treat due to high tumor burden and limited systemic options. This study evaluated the efficacy and safety of drug-eluting bead transarterial chemoembolization (DEB-TACE) combined with intra-arterial infusion of PD-L1 inhibitor TQB2450 and oral Anlotinib as first-line treatment in patients with advanced HCC.

**Methods:**

In this prospective, single-arm phase 2 trial, 31 patients with BCLC stage C HCC received DEB-TACE, transarterial infusion of TQB2450 (1200 mg every 3 weeks), and oral Anlotinib (12 mg/day, 2 weeks on/1 week off). Tumor responses were assessed using mRECIST criteria. Primary endpoint was objective response rate (ORR); secondary endpoints included progression-free survival (PFS), overall survival (OS), and safety.

**Results:**

The ORR was 54.5%, with 3 patients (9.7%) achieving complete response. The median PFS and OS were 5.1 and 9.6 months, respectively. The most common grade ≥3 adverse events were thrombocytopenia (16.1%) and elevated bilirubin (12.9%). Most toxicities were manageable.

**Conclusions:**

This triple-combination therapy demonstrated potentially beneficial antitumor activity and an acceptable safety profile in patients with advanced HCC. These findings support further investigation in randomized controlled trials.

**Clinical trial number:**

Chinese Clinical Trials Database (ChiCTR2200056222).

## 1 Introduction

Hepatocellular carcinoma (HCC) is the sixth most common cancer and the third leading cause of cancer-related mortality globally (1). The majority of patients are diagnosed at Barcelona Clinic Liver Cancer (BCLC) stage C, thereby missing the opportunity for curative treatments such as surgical resection, liver transplantation, or local ablation (2). Advanced hepatocellular carcinoma (HCC) poses significant therapeutic challenges (3). According to the BCLC treatment algorithm, patients with BCLC stage C (advanced-stage HCC) are typically treated with systemic therapies, including tyrosine kinase inhibitors (TKIs) targeting vascular endothelial growth factor receptors (VEGFRs), and immune checkpoint inhibitors (ICIs) directed against programmed cell death protein 1 (PD-1), programmed death-ligand 1 (PD-L1), and cytotoxic T-lymphocyte-associated protein 4 (CTLA-4) pathways, and combinations of these treatments (4–11).

However, given the heterogeneous nature of disease burden in BCLC stage C, including the presence of macrovascular invasion or limited extrahepatic spread, a subset of patients may benefit from locoregional therapies such as TACE (8, 9). TACE, a cornerstone locoregional therapy for advanced HCC, demonstrates superior efficacy in achieving intrahepatic tumor control. Current TACE protocols are classified into two principal modalities based on embolic material composition: conventional TACE (cTACE) utilizing lipiodol emulsions, and drug-eluting bead TACE (DEB-TACE) employing calibrated microspheres with sustained chemotherapeutic release profiles, and DEB-TACE has better efficacy than cTACE (12).

TACE combined with TKIs can inhibit hypoxia-induced tumor neovascularization, thereby reducing the risk of tumor recurrence and progression (13). Anlotinib is a multi-targeted TKI with anti-tumor activity, but it has not been widely used in the treatment of HCC. Studies have shown that Anlotinib can significantly inhibit HCC cell viability, proliferation, colony formation, and promote cell apoptosis *in vitro* (14). Anlotinib combined with TACE and immunosuppressive agents can achieve certain efficacy (15, 16).

Immunotherapy plays an important role in the treatment of tumors (17). A number of studies have confirmed the effectiveness of immunotherapy combined with TACE and targeted drugs in the treatment of HCC. However, although the therapeutic effect has been improved, it is not satisfactory (8, 9).

TQB2450 is a novel humanized anti-PD-L1 IgG1 antibody that has been shown to be effective against a variety of tumors including HCC (18–20). In clinical practice, PD -1 or PD-L1 inhibitors are administered primarily intravenously. Previous studies have investigated the clinical efficacy of intra-arterial infusion of PD-1 inhibitors in HCC, showing positive efficacy and an acceptable safety profile (21, 22). Theoretically, DEB-TACE combined with transarterial infusion of PD-1 inhibitors combined with oral Anlotinib can further improve the efficacy of HCC.

We therefore designed a single-arm, single-center, phase 2 study to evaluate the efficacy and safety of DEB-TACE combined with transarterial infusion of TQB2450 and oral Anlotinib in the treatment of hepatocellular carcinoma.

## 2 Materials and methods

### 2.1 Study design and patients

This study was a single-arm, single-center exploratory study. The study was approved by the ethics committee of our hospital (Ethics number: 2021-281-004). All patients signed informed consent before participating in the study. The study is registered with https://www.chictr.org.cn/, ChiCTR2200056222.

Patients were enrolled if they met all of the following criteria: (1) Initial clinical or pathological diagnosis of hepatocellular carcinoma (HCC); (2) Child-Pugh score ≤ 7; (3) Male or female patients aged 18–75 years; (4) BCLC Stage C; (5) Eastern Cooperative Oncology Group Performance Status (ECOG PS) score of 0–1 and life expectancy >3 months; (6) At least one measurable lesion meeting Response Evaluation Criteria in Solid Tumors version 1.1 (RECIST v1.1) criteria; (7) Laboratory parameters meeting all of the following: Absolute neutrophil count (ANC) ≥1.5×10^9^/L, Platelet count ≥75×10^9^/L, Total bilirubin ≤1.5×upper limit of normal (ULN), Alanine aminotransferase (ALT) and aspartate aminotransferase (AST) ≤2.5×ULN, Creatinine clearance (CrCl) ≥60 mL/min (calculated by Cockcroft-Gault formula or measured), International normalized ratio (INR) or prothrombin time (PT) ≤1.5×ULN.

Exclusion criteria included: (1) Any prior antineoplastic therapy for hepatocellular carcinoma; (2) History of autoimmune diseases; (3) Decompensated cirrhosis (manifested by hepatic encephalopathy, variceal bleeding, or hepatorenal syndrome); (4) Refractory ascites unresponsive to medical therapy; (5) Hypersensitivity to any study drug; (6) Major bleeding events or clinically significant coagulopathy within 3 months before enrollment; (7) Hepatitis B virus (HBV) DNA ≥500 IU/mL; (8) Active hepatitis C infection (HCV antibody-positive with detectable HCV-RNA); (9) Significant cardiopulmonary insufficiency (NYHA Class III/IV heart failure or FEV_1_ <50% predicted); (10) History of other malignant tumors within 5 years (excluding cured non-melanoma skin cancer/carcinoma *in situ*); (11) Complete portal vein occlusion with insufficient collateral circulation (cavernous transformation), and unsuitability for portal vein stenting to restore hepatic inflow.

The enrolled patients were excluded if they met any of the following criteria: (1) Incorrect medication dose or method (the actual exposure dose of the drug was less than 80% or greater than 120% of the prescribed dose); (2) Patients who received chemotherapy, surgery, or investigational drug therapy outside of the protocol during the trial; (3) Patients who did not meet the inclusion criteria and were erroneously enrolled; and (4) Patients who did not receive the medication.

### 2.2 Procedure

All patients received at least one DEB-TACE combined with transarterial infusion of TQB2450 and one cycle of oral Anlotinib. The dosages used for TQB2450 (1200 mg Q3W), DEB-TACE, and Anlotinib (12 mg daily, 2 weeks on/1 week off) were based on prior studies (14–19) and clinical experience. DEB-TACE was then performed on demand, along with transarterial infusion of TQB2450, and when DEB-TACE was not required, TQB2450 was pumped intravenously. TQB2450 was used every 3 weeks. A dose of 1200 mg of TQB2450 was thoroughly mixed with 250 ml of normal saline and infused over two hours.

The procedure is briefly described as follows: After cannulation of the femoral artery, indirect portal venography and celiac angiography were performed. Subsequently, TQB2450 perfusion was administered in the proper hepatic artery via a microcatheter. Following this step, the intrahepatic lesions were super-selectively injected with drug-eluting microspheres loaded with pirarubicin (40 mg, polyvinyl alcohol embolization microspheres, blue, diameter 300–500 μm, 2 ml; Jiangsu Hengrui Pharmaceutical Co., Ltd.). The endpoint of embolization was defined as the significant slowing or complete cessation of blood flow in the tumor-feeding artery.

**Figure 1:**
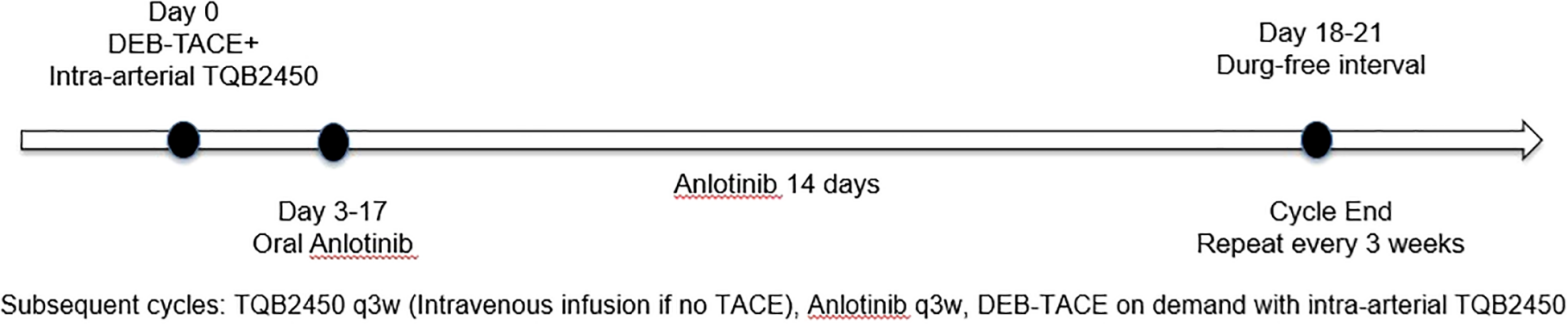
Schematic diagram of the timing of intervention measures.

Oral Anlotinib (CP Tianqing Pharmaceutical Group Co., Ltd., 12 mg/tablet) was initiated on the third day after DEB-TACE. The dosing regimen consisted of 12 mg once daily for 14 days, followed by a 7-day drug-free interval. The schematic diagram of the timing of intervention measures shown in **Figure 1**. For Anlotinib dose modification, a tiered de-escalation protocol was implemented: initial dose reduction to 10 mg/day followed by 8 mg/day if 12 mg/day proved intolerable. Permanent discontinuation occurred upon persistent intolerance. In addition to that, study cessation criteria included any of the following: confirmed disease progression, unmanageable toxicity, initiation of alternative antineoplastic regimens, voluntary withdrawal, mortality, or other protocol-specified termination events. After discontinuation of the study, TQB2450 and Anlotinib were discontinued, the patient transitioned to survival monitoring without any intervention in the patient’s other treatments.

The on-treatment time was defined as the duration from patient enrollment to study discontinuation, death, or the follow-up cutoff date. The follow-up time refers to the interval from study discontinuation to death or the end of the follow-up period. During this period, patients were no longer administered TQB2450 or Anlotinib; however, the patients remained eligible for other clinically appropriate treatments, such as TACE, ablation, radiotherapy, or combinations of local therapy with targeted and/or immunotherapy.

### 2.3 Follow−up and endpoints assessment

Protocol-mandated evaluations were performed every 3 weeks during the treatment period, including comprehensive laboratory assessments such as complete blood count, hepatic and renal function tests, coagulation profile, alpha-fetoprotein levels, fecal occult blood test, and urinalysis. Imaging (either multiphase contrast-enhanced CT or dynamic contrast-enhanced MRI) was conducted every 6 weeks (every two treatment cycles). All imaging studies were independently reviewed in a blinded manner by two senior radiologists. In cases of discordance, a third radiologist with over 15 years of experience adjudicated the findings, and the consensus was deemed final. Tumor response was assessed every 6 weeks according to the modified Response Evaluation Criteria in Solid Tumors (mRECIST) (23) and categorized as complete response (CR), partial response (PR), stable disease (SD), or progressive disease (PD).

The primary endpoint was objective response rate (ORR, defined as the proportion of patients achieving CR or PR). Secondary endpoints included progression-free survival (PFS, defined as the time from randomization to radiologically confirmed disease progression or death from any cause), overall survival (OS, defined as the time from randomization to death from any cause) and safety. All treatment-related adverse events (TRAEs) were systematically recorded and graded according to the Common Terminology Criteria for Adverse Events (CTCAE), version 4.0 (24).

### 2.4 Statistical analysis

All analyses were performed using R (version 4.1.2; R Foundation for Statistical Computing, Vienna, Austria). Continuous variables were expressed as mean ± standard deviation (SD) when normally distributed or median with interquartile range (IQR) [M (Q1, Q3)] for non-normally distributed data. Survival curves were generated using the Kaplan-Meier method, with median overall survival (mOS) and median progression-free survival (mPFS) as key metrics.

## 3 Results

### 3.1 Patient characteristics

**Figure 2:**
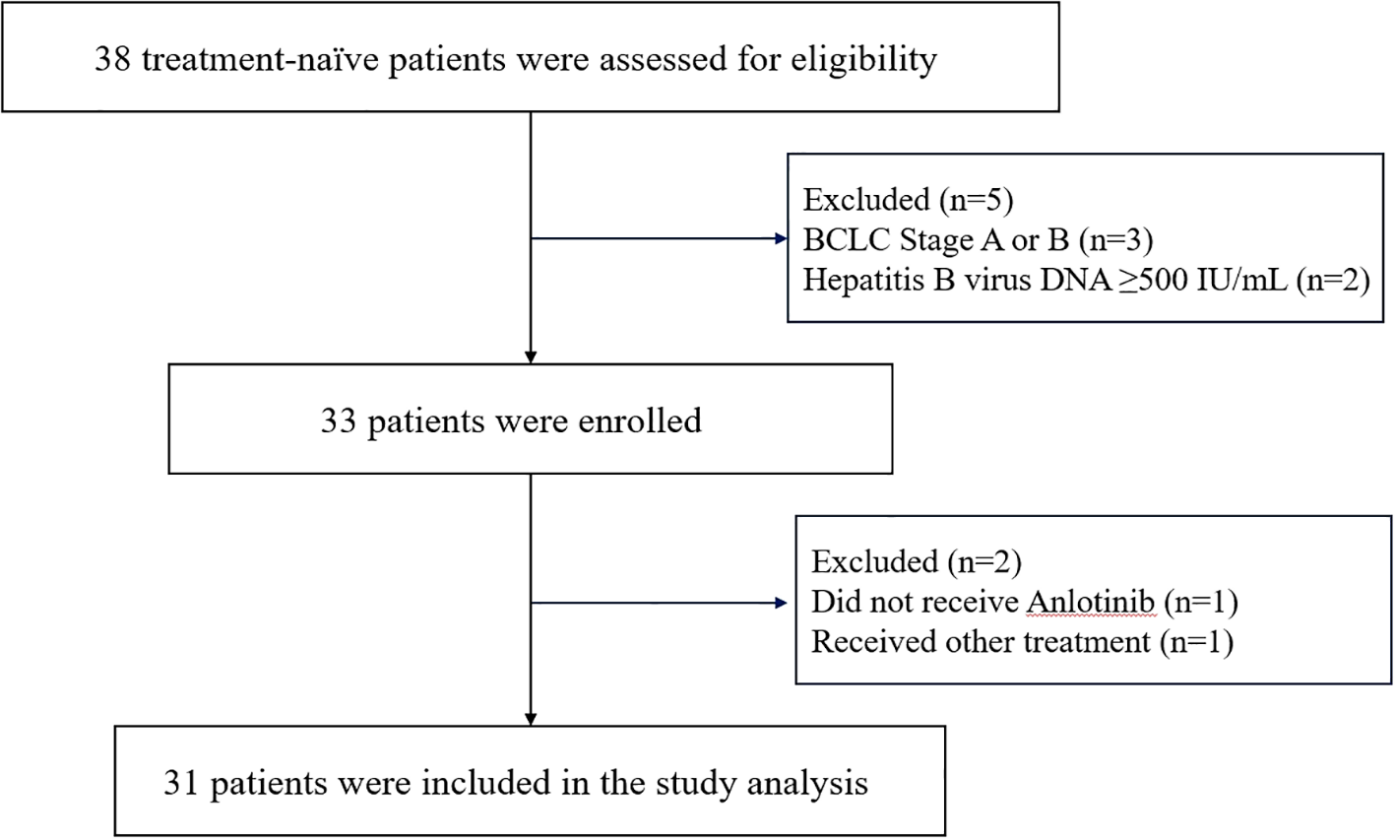
Patient flow chart.

From March 3, 2022 to March 12, 2024, a total of 38 patients were screened. Among them, 3 patients were excluded due to not meeting the staging criteria, and 2 patients were excluded based on their hepatitis B virus DNA levels. Thus, 33 patients were initially enrolled in the study. During the subsequent treatment phase, 1 patient did not receive Anlotinib therapy, and another was excluded due to receiving additional radiotherapy during the study period (**Figure 2**). Follow-up was conducted up to September 1, 2024, with a final cohort of 31 patients included in the analysis. Baseline characteristics are summarized in **Table 1**. The median age was 55 years, and 22 patients (71.0%) were male. A total of 30 patients (96.8%) had chronic hepatitis B infection. Most patients (83.9%) had an ECOG performance status score of 0. Six patients (19.4%) had more than three intrahepatic lesions. The mean maximum diameter of the largest tumor was 87.97 ± 32.57 mm, while the mean maximum diameter of all intrahepatic lesions was 143.97 ± 55.32 mm. Twenty-five patients (80.6%) presented with varying degrees of portal vein tumor thrombus (PVTT), and 6 patients (19.4%) had inferior vena cava tumor thrombus (IVCTT). Fifteen patients (48.4%) exhibited extrahepatic metastases. All patients received between 1 and 6 sessions of DEB-TACE, with a median of 4 sessions.

**Table 1:** Baseline characteristics.

Variables	n (%)
Age, years	
Median [IQR]	55 [49, 62]
Gender	
Female	9 (29.0%)
Male	22 (71.0%)
Etiology	
HBV	30 (96.8%)
HCV	1 (3.2%)
ECOG performance score	
0	26 (83.9%)
1	5 (16.1%)
Child–Pugh Score	
5	24 (77.4%)
6	6 (19.4%)
7	1 (3.2%)
AFP	
<400 ng/ml	16 (51.6%)
≥400 ng/ml	15 (48.4%)
Intrahepatic tumor number	
≤3	25 (80.6%)
>3	6 (19.4%)
Maximum intrahepatic tumor size, mm	
Mean (SD)	87.97 (32.57)
Median [IQR]	86 [65, 109]
Total intrahepatic tumor size, mm	
Mean (SD)	143.97 (55.32)
Median [IQR]	145 [98, 186]
PVTT	
Vp2	3 (9.7%)
Vp3	18 (58.1%)
Vp4	4 (12.9%)
Absent	6 (19.3%)
IVCTT	
IVC invasion	6 (19.4%)
Absent	25 (80.6%)
Extrahepatic metastasis	
Present	15 (48.4%)
Absent	16 (51.6%)
Number of DEB-TACE procedures	
Median [IQR]	4 [2, 4]

IQR, Interquartile range; ECOG, Eastern Cooperative Oncology Group; AFP, Alpha-fetoprotein; SD, Standard deviation; PVTT, Portal vein tumor thrombus; IVCTT, Inferior vena cava tumor thrombus; IVC, Inferior vena cava; DEB-TACE, Drug-eluting bead- transarterial chemoembolization.

### 3.2 Efficacy

**Figure 3:**
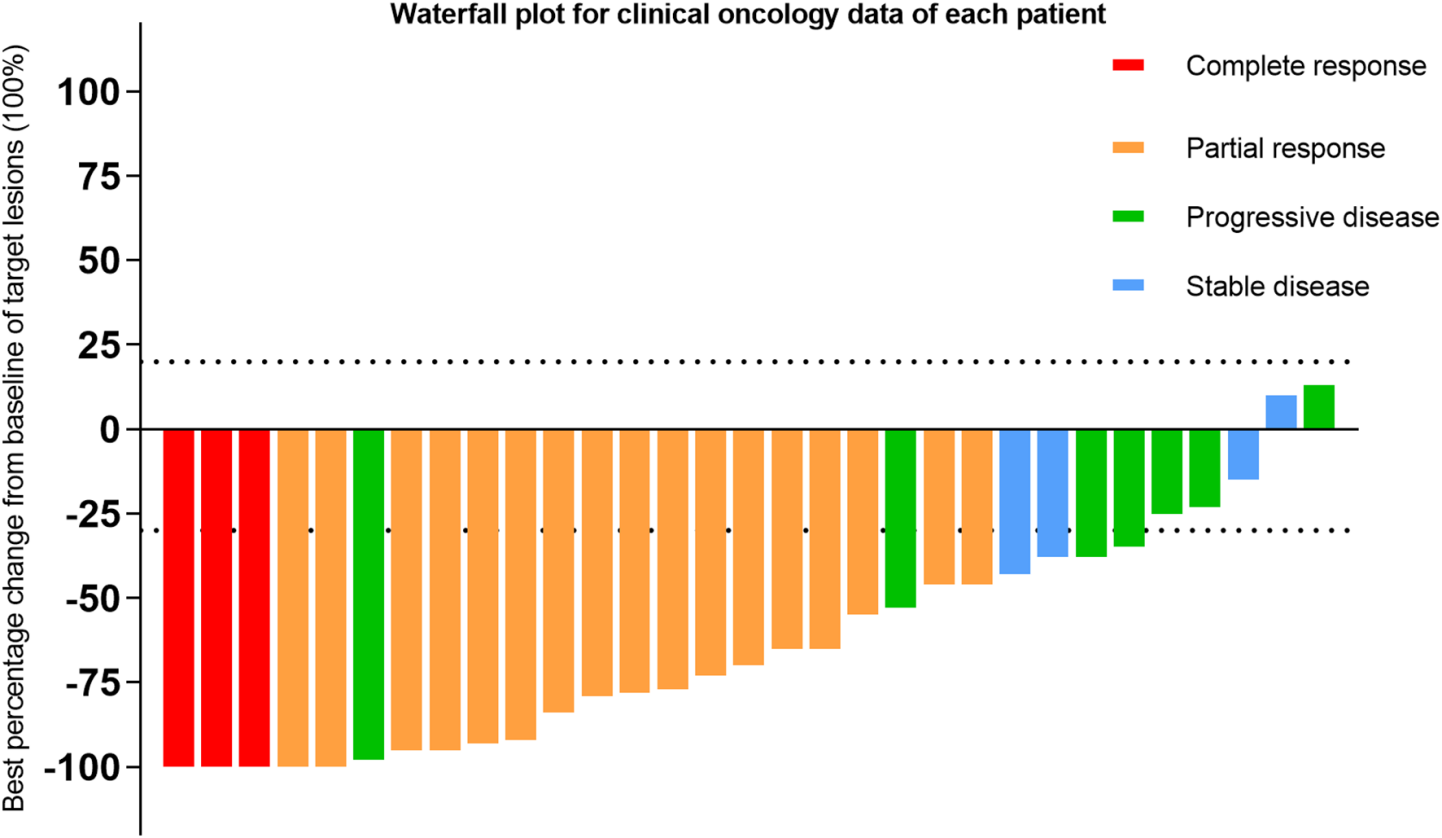
Waterfall plot for clinical oncology data of each patient.

**Figure 4:**
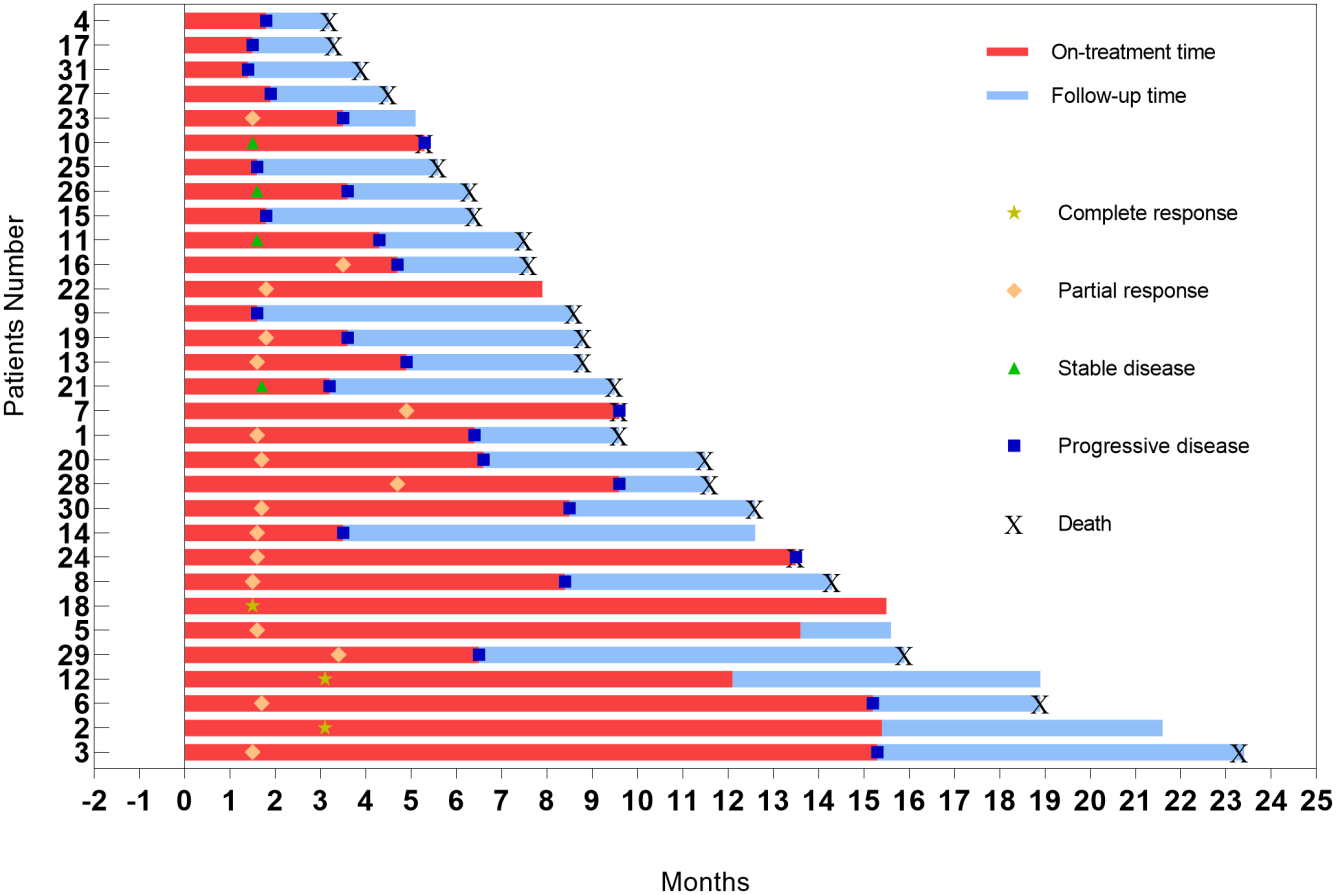
Swimmer’s plot of the comprehensive clinical trajectory of patients in this trial.

The correlation between the optimal percentage change in hepatic target lesions and the overall assessment according to the mRECIST criteria is presented in **Figure 3**. Among the patients, 29 exhibited varying degrees of tumor shrinkage in their liver target lesions, with 3 achieving a CR. The entire study timeline, including key clinical milestones for all patients, is illustrated in **Figure 4**. According to the mRECIST criteria, the best overall tumor response yielded an objective response rate (ORR) of 54.5% and a disease control rate (DCR) of 67.4%. Notably, among the 3 patients (9.7%) who achieved CR in their target lesions, the overall response assessment also confirmed CR (**Table 2**), one of the 3 patients was still receiving maintenance treatment with TQB2450 and anlotinib at the end of the study.

**Table 2:** Best tumor response rates assessed by mRECIST.

Overall response	Investigators assessed by mRECIST n (%)
Complete response	3 (9.7%)
Partial response	17 (54.8%)
Stable disease	4 (12.9%)
Progressive disease	7 (22.6%)
Disease control rate	24 (67.4%)
Objective response rate	20 (54.5%)

**Figure 5:**
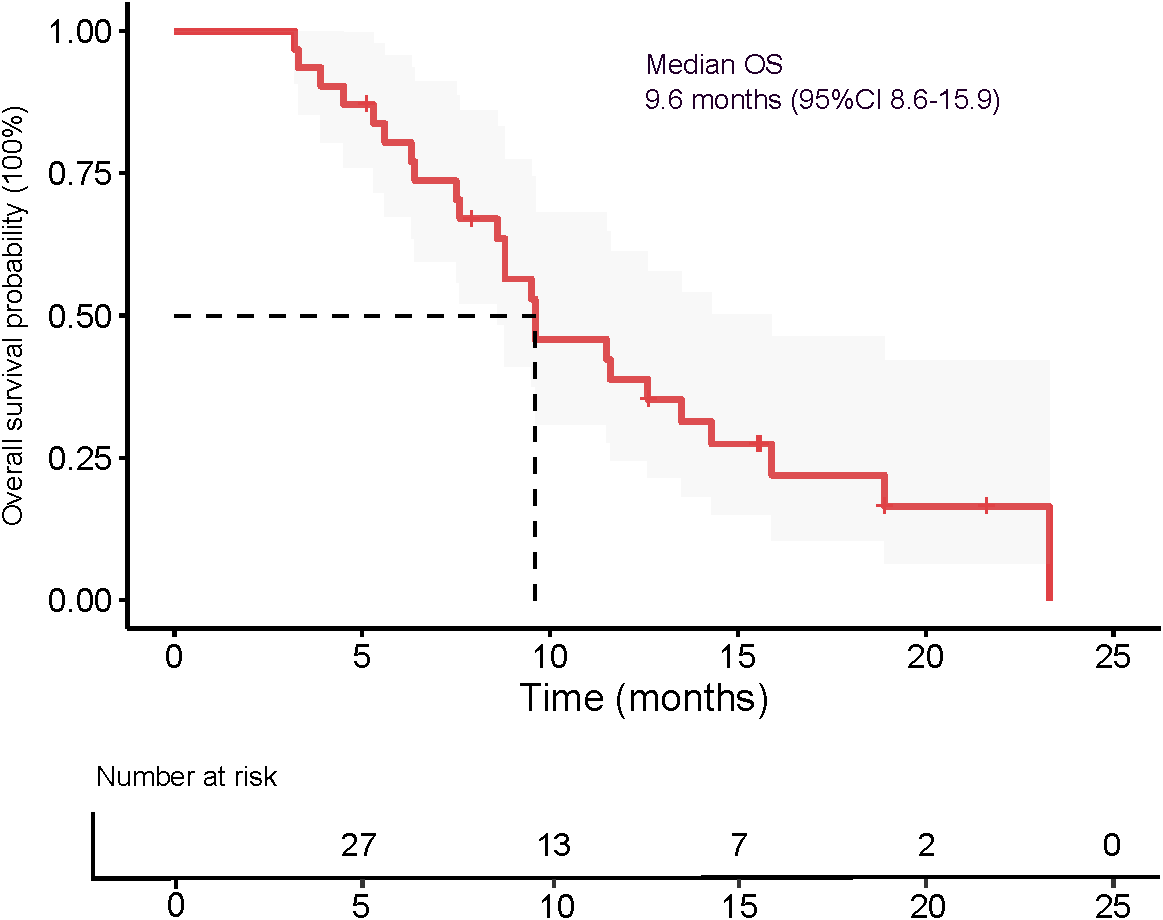
Kaplan-Meier survival curves estimate overall survival in all patients.

**Figure 6:**
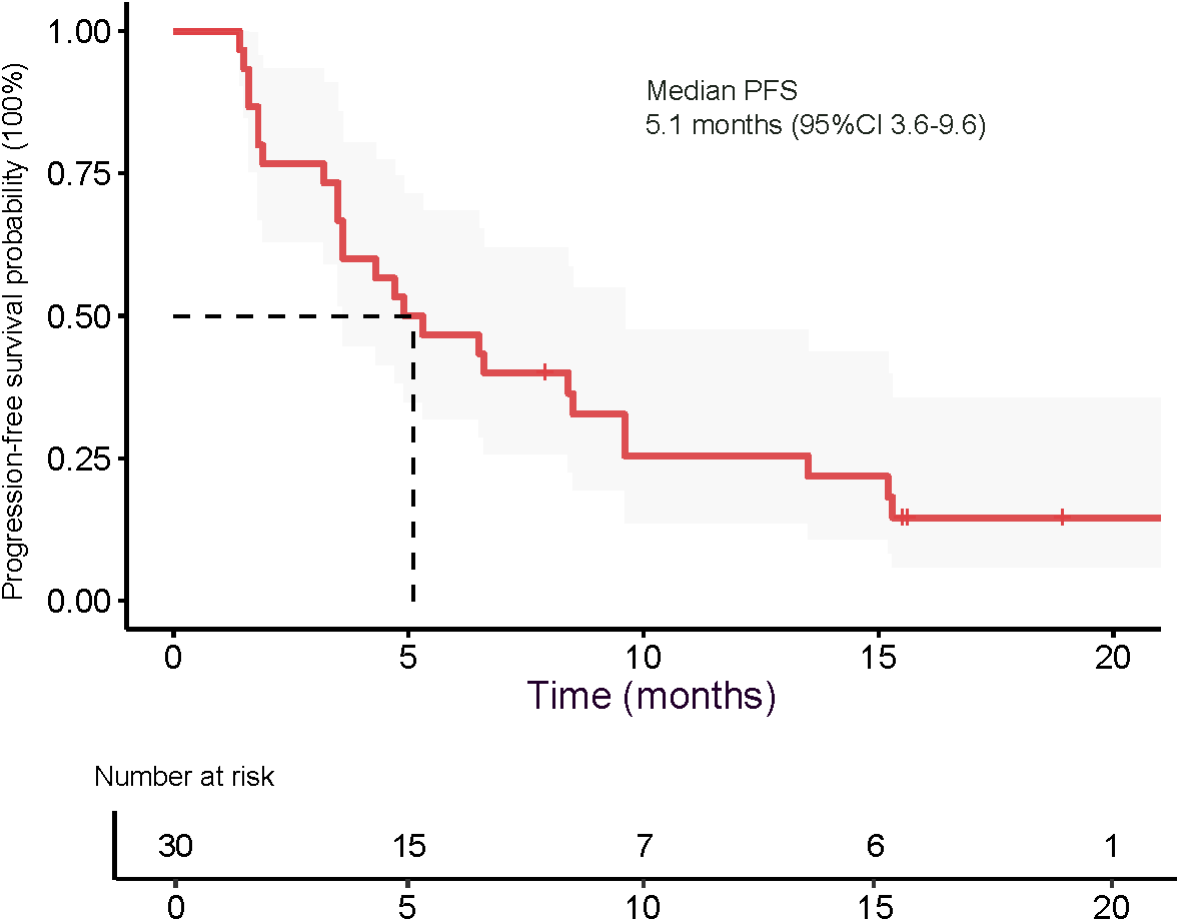
Kaplan-Meier survival curves estimate progression-free survival in all patients.

The median OS was 9.6 months (95% CI 8.6–15.9), as presented in **Figure 5**. The 3-month OS rate was 100%, the 6-month OS rate was 77.4% (24/31), and the 12-month OS rate was 22.6% (7/31). The median PFS was 5.1 months (95% CI 3.6–9.6), as shown in **Figure 6**. The 3-month PFS rate was 77.4% (24/31), the 6-month PFS rate was 48.4% (15/31), and the 12-month PFS rate was 22.6% (7/31).

### 3.3 Safety

Treatment-related adverse events (TRAE) were predominantly hepatic dysfunction and hematologic toxicity. The most common grade ≥3 adverse events included thrombocytopenia (16.1%) and elevated serum bilirubin (12.9%), whereas the most frequently reported events of any grade were abdominal pain (90.3%), hypoalbuminemia (77.4%), fever (74.2%), and elevated transaminase levels (AST 80.6%; ALT 71.0%). Although TACE-related complications were common, most were classified as grade 1–2. Notably, typical antiangiogenic therapy-associated adverse events were observed, including hypertension (54.8%), ascites (58.1%), and gastrointestinal bleeding (19.4%), with one case (3.2%) of grade 3 gastrointestinal bleeding. Mild immune-related adverse events, such as hypothyroidism (22.6%) and proteinuria (19.4%), were also recorded (**Table 3**).

**Table 3:** Treatment-related adverse events.

Categories	All grades, n (%)	Grades 1-2, n (%)	≥Grades 3 n (%)
Decreased white blood cell count	13 (41.9%)	11 (35.5%)	2 (6.5%)
Decreased neutrophil count	6 (19.4%)	6 (19.4%)	0 (0%)
Decreased platelet count	16 (51.6%)	11 (35.5%)	5 (16.1%)
Anemia	5 (16.1%)	5 (16.1%)	0 (0%)
Elevated aspartate aminotransferase	25 (80.6%)	23 (74.2%)	2 (6.5%)
Elevated alanine aminotransferase	22 (71.0%)	21 (67.7%)	1 (3.2%)
Hypoalbuminemia	24 (77.4%)	24 (77.4%)	0 (0%)
Blood bilirubin increased	19 (61.3%)	15 (48.4)	4 (12.9)
Proteinuria	6 (19.4%)	6 (19.4%)	0 (0%)
Hypothyroidism	7 (22.6%)	7 (22.6%)	0 (0%)
Hyperglycemia	14 (45.2%)	14 (45.2%)	0 (0%)
Hypertension	17 (54.8%)	17 (54.8%)	0 (0%)
Gastrointestinal hemorrhage	6 (19.4%)	5 (16.1%)	1 (3.2%)
Fatigue	9 (29.0%)	9 (29.0%)	0 (0%)
Nausea	12 (38.7%)	12 (38.7%)	0 (0%)
Hand-foot syndrome	7 (22.6%)	7 (22.6%)	0 (0%)
Diarrhea	3 (9.7%)	3 (9.7%)	0 (0%)
Abdominal pain	28 (90.3%)	28 (90.3%)	0 (0%)
Fever	23 (74.2%)	23 (74.2%)	0 (0%)
Ascites	18 (58.1%)	16 (51.6%)	2 (6.5%)

## 4 Discussion

In this single-arm, phase 2 study, we evaluated the efficacy and safety of DEB-TACE combined with transarterial infusion of the PD-L1 inhibitor TQB2450 and oral administration of Anlotinib in patients with advanced HCC. The combination therapy yielded an ORR of 54.5% and a DCR of 67.4% based on mRECIST criteria. Notably, 9.7% of patients achieved a complete response. The median PFS was 5.1 months, and the median OS reached 9.6 months, with a 6-month OS rate of 77.4%. These results suggest that this triple-combination strategy may offer clinically meaningful antitumor activity in a population with BCLC stageC HCC. The treatment was generally well tolerated, with manageable toxicities primarily related to hepatic dysfunction, hematologic abnormalities, and expected class-specific adverse events from antiangiogenic and immune therapies.

The treatment of advanced HCC remains challenging. Since the introduction of Sorafenib, targeted therapies have rapidly evolved. Kudo et al. (5) demonstrated that Lenvatinib exhibited therapeutic efficacy comparable to that of Sorafenib in patients with advanced HCC. Anlotinib, a TKI, has been shown to serve as an effective adjuvant therapy for high-risk HCC patients following surgical resection, offering a recurrence prevention effect similar to that of TACE (25). Furthermore, its combination with TACE and radiofrequency ablation (RFA) has shown significant efficacy in treating HCC with PVTT (15). Meanwhile, with the rapid advancement of immunotherapy, several immune checkpoint inhibitors have been approved for the treatment of advanced HCC (26). Recently, some researchers have reported promising outcomes in treating metastatic melanoma through intra-arterial infusion of PD-1 antibodies (27, 28). The rationale behind this approach is that delivering immune agents directly to the tumor via arterial infusion can increase local drug concentration and enhance PD-1 receptor inhibition. However, these two studies, along with two more recent investigations (21, 22), have explored the use of transarterial infusion of PD-1 inhibitors in cancer therapy. TQB2450 is a novel PD-L1 receptor inhibitor. In a phase I study, it was confirmed that its combination with TKI drugs had good safety in the treatment of advanced hepatocellular carcinoma (20). However, two other studies (18, 19), TQB2450 combined with anlotinib in the treatment of non-small cell lung cancer, confirmed the safety of the combination of these two drugs.

Therefore, TACE/DEB-TACE combined with targeted therapy and immunotherapy is increasingly favored for the treatment of advanced HCC. Cai et al. (8) reported that TACE in combination with Lenvatinib and a PD-1 inhibitor achieved a median OS of 16.9 months and a median PFS of 7.3 months. Compared with TACE alone combined with Lenvatinib, no significant increase in TAREs was observed. The median OS and PFS in this study were higher than those in our study, which may be attributed to the higher proportion of patients with PVTT in our cohort (80.0% vs. 36.6%). Chen et al. (9) conducted a retrospective study showing that TACE combined with Lenvatinib and pembrolizumab resulted in a median survival of 18.1 months and a PFS of 9.2 months. The rates of hypertension, nausea, and rash were higher with the combination of TACE and Lenvatinib compared to TACE alone. The results of this study demonstrated favorable efficacy, with triple therapy showing particularly significant benefits in patients with PD-L1 expression, despite an increased incidence of treatment-related adverse events, which remained manageable. Another study evaluating TACE plus durvalumab with or without bevacizumab in patients with unresectable HCC showed a median PFS of 27.9 months with triple therapy, compared to 15 months with targeted and immune therapy alone, and only 10 months with durvalumab monotherapy (29). This finding further underscores the therapeutic advantages of combining TACE with targeted and immunotherapeutic approaches.

The CHANCE2201 study (NCT05332821) (30) demonstrated that TACE combined with ICIs and anti-VEGF agents significantly improved overall survival, progression-free survival, and objective response rates in advanced HCC patients. Similarly, the LEAP-012 trial (NCT04246177) (31) showed that TACE combined with lenvatinib and pembrolizumab significantly prolonged progression-free survival in unresectable, non-metastatic HCC. These findings support the efficacy of triple combination therapy integrating locoregional, immunotherapeutic, and antiangiogenic treatments in advanced hepatocellular carcinoma.Recent studies have demonstrated that transarterial infusion of PD-1 inhibitors is both safe and effective. Shen et al. (21) employed TACE combined with intra-arterial infusion of Sintilimab and Bevacizumab for the treatment of advanced HCC, achieving a median PFS of 6 months and a median OS of 12.2 months. In contrast, Mu et al. (22) utilized Hepatic arterial infusion chemotherapy (HAIC) in combination with arterial infusion of Camrelizumab and oral Apatinib for advanced HCC, reporting an ORR of 44%, a median OS of 8.87 months, and a median PFS of 4.87 months. Our study employed DEB-TACE combined with intra-arterial infusion of TQB2450 and oral anlotinib for the treatment of advanced HCC, yielding a median OS of 9.6 months and a median PFS of 5.1 months. These outcomes were comparable to those reported in the aforementioned two studies, but inferior to those observed in other studies utilizing intravenous immune agents. Potential explanations for this discrepancy may include the limited sample size in our study, which could introduce bias. Further large-scale studies are warranted to confirm these findings.

The majority of TAREs in this study were grade 1–2, with grade ≥3 adverse events being rare, primarily consisting of hematological reactions. Given that HCC patients often have a background of cirrhosis and hypersplenism, many already exhibit reduced white blood cell counts and platelet levels prior to treatment. Additionally, the main TAREs observed were embolization syndrome symptoms, such as pain and fever, commonly associated with DEB-TACE. Notably, the relatively low incidence of adverse events related to targeted agents may be attributed to the pharmacological characteristics of Anlotinib itself, as well as its dosing schedule—2 weeks of oral administration followed by 1 week off—and the availability of three different dosage strengths, which allow for dose titration based on individual patient tolerance. Overall, the combination of DEB-TACE with intra-arterial infusion of TQB2450 and oral Anlotinib demonstrated a favorable safety profile and was considered safe and well-tolerated.

This study has several limitations. First, it was a single-center, single-arm phase II trial with a relatively small sample size, which may limit the generalizability of the findings. The absence of a control group precludes direct comparison with standard treatment regimens, and potential selection bias cannot be ruled out. Second, although the combination strategy showed potential clinical efficacy, the median OS and PFS remain modest, possibly reflecting the advanced disease stage of the enrolled population, most of whom presented with portal vein tumor thrombus and extrahepatic metastases. Third, the follow-up period was relatively short, limiting the assessment of long-term survival benefits and delayed immune-related adverse events. Fourth, all patients received DEB-TACE with variable numbers of sessions. This heterogeneity in TACE frequency might have influenced treatment efficacy and survival outcomes, and should be interpreted with caution. Lastly, the use of intra-arterial delivery of PD-L1 inhibitors, while theoretically advantageous, warrants further investigation to optimize dosing, scheduling, and pharmacokinetics in comparison to standard intravenous administration. Future research will focus on conducting prospective, multicenter, randomized controlled trials with larger sample sizes to further validate the efficacy and safety of this combined treatment regimen. Additionally, biomarker-based patient stratification and investigation into the underlying immunologic and molecular mechanisms may help identify patient populations most likely to benefit from the therapy, as well as inform optimized treatment sequencing and dosing strategies.

## 5 Conclusion

In conclusion, DEB-TACE combined with transarterial infusion of TQB2450 and oral Anlotinib demonstrated potentially beneficial antitumor activity and an acceptable safety profile in patients with advanced hepatocellular carcinoma. This triple-modality approach may offer a synergistic therapeutic benefit by integrating locoregional control, anti-angiogenic inhibition, and immune checkpoint blockade. Further large-scale, randomized controlled trials are warranted to validate these findings and determine the optimal treatment strategy for this challenging patient population.

## Data Availability

The raw data supporting the conclusions of this article will be made available by the authors, without undue reservation.

## References

[1] Sung H Ferlay J Siegel RL Laversanne M Soerjomataram I Jemal A. Global cancer statistics 2020: GLOBOCAN estimates of incidence and mortality worldwide for 36 cancers in 185 countries. CA Cancer J Clin. (2021) 71:209–49. doi: 10.3322/caac.21660, PMID: 33538338

[2] Forner A Reig M Bruix J. Hepatocellular carcinoma. Lancet. (2018) 391:1301–14. doi: 10.1016/S0140-6736(18)30010-2, PMID: 29307467

[3] Llovet JM De Baere T Kulik L Haber PK Greten TF Meyer T. Locoregional therapies in the era of molecular and immune treatments for hepatocellular carcinoma. Nat Rev Gastroenterol Hepatol. (2021) 18:293–313. doi: 10.1038/s41575-020-00395-0, PMID: 33510460

[4] Raoul JL Forner A Bolondi L Cheung TT Kloeckner R de Baere T. Updated use of TACE for hepatocellular carcinoma treatment: How and when to use it based on clinical evidence. Cancer Treat Rev. (2019) 72:28–36. doi: 10.1016/j.ctrv.2018.11.002, PMID: 30447470

[5] Kudo M Finn RS Qin S Han KH Ikeda K Piscaglia F. Lenvatinib versus sorafenib in first-line treatment of patients with unresectable hepatocellular carcinoma: a randomised phase 3 non-inferiority trial. Lancet. (2018) 391:1163–73. doi: 10.1016/S0140-6736(18)30207-1, PMID: 29433850

[6] Ren Z Xu J Bai Y Xu A Cang S Du C. Sintilimab plus a bevacizumab biosimilar (IBI305) versus sorafenib in unresectable hepatocellular carcinoma (ORIENT-32): a randomised, open-label, phase 2–3 study. Lancet Oncol. (2021) 22:977–90. doi: 10.1016/S1470-2045(21)00252-7, PMID: 34143971

[7] Yau T Park JW Finn RS Cheng AL Mathurin P Edeline J. Nivolumab versus sorafenib in advanced hepatocellular carcinoma (CheckMate 459): a randomised, multicentre, open-label, phase 3 trial. Lancet Oncol. (2022) 23:77–90. doi: 10.1016/S1470-2045(21)00604-5, PMID: 34914889

[8] Cai M Huang W Huang J Shi W Guo Y Liang L. Transarterial chemoembolization combined with lenvatinib plus PD-1 inhibitor for advanced hepatocellular carcinoma: A retrospective cohort study. Front Immunol. (2022) 13:848387. doi: 10.3389/fimmu.2022.848387, PMID: 35300325 PMC8921060

[9] Chen S Wu Z Shi F Mai Q Wang L Wang F. Lenvatinib plus TACE with or without pembrolizumab for the treatment of initially unresectable hepatocellular carcinoma harbouring PD-L1 expression: a retrospective study. J Cancer Res Clin Oncol. (2022) 148:2115–25. doi: 10.1007/s00432-021-03767-4, PMID: 34453221 PMC9293824

[10] Rimassa L Finn RS Sangro B. Combination immunotherapy for hepatocellular carcinoma. J Hepatol. (2023) 79:506–15. doi: 10.1016/j.jhep.2023.03.003, PMID: 36933770

[11] Sutanto H Adytia G Elisa E Maimunah U. Advances in transarterial chemoembolization for hepatocellular carcinoma: Integration with systemic therapies and emerging treatment strategies. Cancer Pathog Ther. (2025) 12:2493457. doi: 10.1016/j.cpt.2025.04.004 PMC12639617 41280241

[12] Bargellini I Lorenzoni V Lorenzoni G Scalise P Andreozzi G Bozzi E. Duration of response after DEB-TACE compared to lipiodol-TACE in HCC-naïve patients: a propensity score matching analysis. Eur Radiol. (2021) 31:7512–22. doi: 10.1007/s00330-021-07905-x, PMID: 33871708 PMC8452560

[13] Geschwind JF Gholam PM Goldenberg A Mantry P Martin RC Piperdi B. Use of transarterial chemoembolization (TACE) and sorafenib in patients with unresectable hepatocellular carcinoma: US regional analysis of the GIDEON registry. Liver Cancer. (2016) 5:37–46. doi: 10.1159/000367757, PMID: 26989658 PMC4789900

[14] He C Wu T Hao Y. Anlotinib induces hepatocellular carcinoma apoptosis and inhibits proliferation via Erk and Akt pathway. Biochem Biophys Res Commun. (2018) 503:3093–9. doi: 10.1016/j.bbrc.2018.08.098, PMID: 30146257

[15] Wang J Li X Wang F Shi D Zhang J. Anlotinib followed by transarterial chemoembolization and radiofrequency ablation is a safe and effective initial treatment for hepatocellular carcinoma patients with portal vein tumor thrombus: A retrospective case series study. J Cancer Res Ther. (2021) 17:619–24. doi: 10.4103/jcrt.JCRT_1253_20, PMID: 34269290

[16] Chen XQ Zhao YX Zhang CL Wang XT Zhang X Chen X. Effectiveness and safety of anlotinib with or without PD-1 blockades in the treatment of patients with advanced primary hepatocellular carcinoma: A retrospective, real-world study in China. Drug Des Devel Ther. (2022) 16:1483–93. doi: 10.2147/DDDT.S358092, PMID: 35607597 PMC9123907

[17] Igarashi Y Sasada T. Cancer vaccines: toward the next breakthrough in cancer immunotherapy. J Immunol Res. (2020) 2020:5825401. doi: 10.1155/2020/5825401, PMID: 33282961 PMC7685825

[18] Zhang W Wang J Wang Q Cheng Y Yang L Li Y. A randomized double-blind trial of TQB2450 with or without anlotinib in pretreated driver-negative non-small cell lung cancer. Lung Cancer. (2023) 184:107353. doi: 10.1016/j.lungcan.2023.107353, PMID: 37647728

[19] Tan Z Wu Y Fan Z Gao T Guo W Bai C. Anlotinib plus TQB2450, a PD-L1 antibody, in patients with advanced alveolar soft part sarcoma: A single-arm, phase II trial. Clin Cancer Res. (2024) 30:5577–83. doi: 10.1158/1078-0432.CCR-24-2444, PMID: 39453774

[20] Ning T Li D Deng T Bai Y Chen Y Wang Z. Anti-PD-L1 antibody TQB2450 combined with tyrosine kinase receptor inhibitor AL2846 for immunotherapy-refractory advanced hepatocellular carcinoma and esophageal squamous cell carcinoma: A prospective phase 1b cohort study. Cancer. (2024) 130:3137–46. doi: 10.1002/cncr.35377, PMID: 38781433

[21] Shen L Cao F Liu Y Nuerhashi G Lin L Tan H. Hepatic artery infusion of FOLFOX chemotherapy plus camrelizumab combined with sorafenib for advanced hepatocellular carcinoma in Barcelona Clinic Liver Cancer stage C (Double-IA-001): a phase II trial. BMC Med. (2025) 23:275. doi: 10.1186/s12916-025-04110-1, PMID: 40346494 PMC12065160

[22] Mu MY Chen ZX Cao YZ Fu XB Qiu LJ Qi H. Transarterial chemoembolization combined with intra-arterial infusion of sintilimab and bevacizumab for advanced hepatocellular carcinoma: a phase 2 study. Cancer Lett. (2025) 628:217851. doi: 10.1016/j.canlet.2025.217851, PMID: 40472923

[23] Lencioni R Llovet JM. Modified RECIST (mRECIST) assessment for hepatocellular carcinoma. Semin Liver Dis. (2010) 30:52–60. doi: 10.1055/s-0030-1247132, PMID: 20175033 PMC12268942

[24] Chen AP Setser A Anadkat MJ Cotliar J Olsen EA Garden BC. Grading dermatologic adverse events of cancer treatments: the Common Terminology Criteria for Adverse Events Version 4.0. J Am Acad Dermatol. (2012) 67:1025–39. doi: 10.1016/j.jaad.2012.02.010, PMID: 22502948

[25] Wang J Xiang X Shi Z Zhang H Zhang Q Liu Z. Efficacy and safety of anlotinib as an adjuvant therapy in hepatocellular carcinoma patients with a high risk of postoperative recurrence. Chin J Cancer Res. (2023) 35:399–407. doi: 10.21147/j.issn.1000-9604.2023.04.06, PMID: 37691893 PMC10485915

[26] Zhou J Sun H Wang Z Cong W Zeng M Zhou W. Guidelines for the diagnosis and treatment of primary liver cancer (2022 edition). Liver Cancer. (2023) 12:405–44. doi: 10.1159/000530495, PMID: 37901768 PMC10601883

[27] Shen L Qi H Chen S Cao F Xie L Wu Y. Cryoablation combined with transarterial infusion of pembrolizumab (CATAP) for liver metastases of melanoma: an ambispective, proof-of-concept cohort study. Cancer Immunol Immunother. (2020) 69:1713–24. doi: 10.1007/s00262-020-02566-z, PMID: 32333081 PMC7413875

[28] Qianqi C Yan Z Yueqiang T Jiangman D Xiaohong F Qiming Z. Efficacy and safety of transarterial infusion of anti-PD-1 in the treatment of advanced or metastatic acral and mucosal melanomas. Cell Mol Biol (Noisy-le-grand). (2022) 67:263–8. doi: 10.14715/cmb/2021.67.5.36, PMID: 35818244

[29] Sangro B Kudo M Erinjeri JP Qin S Ren Z Chan SL. Durvalumab with or without bevacizumab with transarterial chemoembolisation in hepatocellular carcinoma (EMERALD-1): a multiregional, randomised, double-blind, placebo-controlled, phase 3 study. Lancet. (2025) 405:216–32. doi: 10.1016/S0140-6736(24)02551-0, PMID: 39798579 PMC12282661

[30] Jin ZC Chen JJ Zhu XL Duan XH Xin YJ Zhong BY. Immune checkpoint inhibitors and anti-vascular endothelial growth factor antibody/tyrosine kinase inhibitors with or without transarterial chemoembolization as first-line treatment for advanced hepatocellular carcinoma (CHANCE2201): a target trial emulation study. EClinicalMedicine. (2024) 72:102622. doi: 10.1016/j.eclinm.2024.102622, PMID: 38745965 PMC11090892

[31] Kudo M Ren Z Guo Y Han G Lin H Zheng J. Transarterial chemoembolisation combined with lenvatinib plus pembrolizumab versus dual placebo for unresectable, non-metastatic hepatocellular carcinoma (LEAP-012): a multicentre, randomised, double-blind, phase 3 study. Lancet. (2025) 405:203–15. doi: 10.1016/S0140-6736(24)02575-3, PMID: 39798578

